# Supplementary material for: The Anti-Inflammatory Effects of Cannabis sativa Extracts on LPS-Induced Cytokines Release in Human Macrophages
Source: Molecules. 2023 Jun 25;28(13):4991. doi: 10.3390/molecules28134991 (PMC10343240; doi:10.3390/molecules28134991)
Supplement: Supplementary file 1 [file molecules-28-04991-s001.zip › molecules-2416686-supplementary.pptx]

## Slide 1
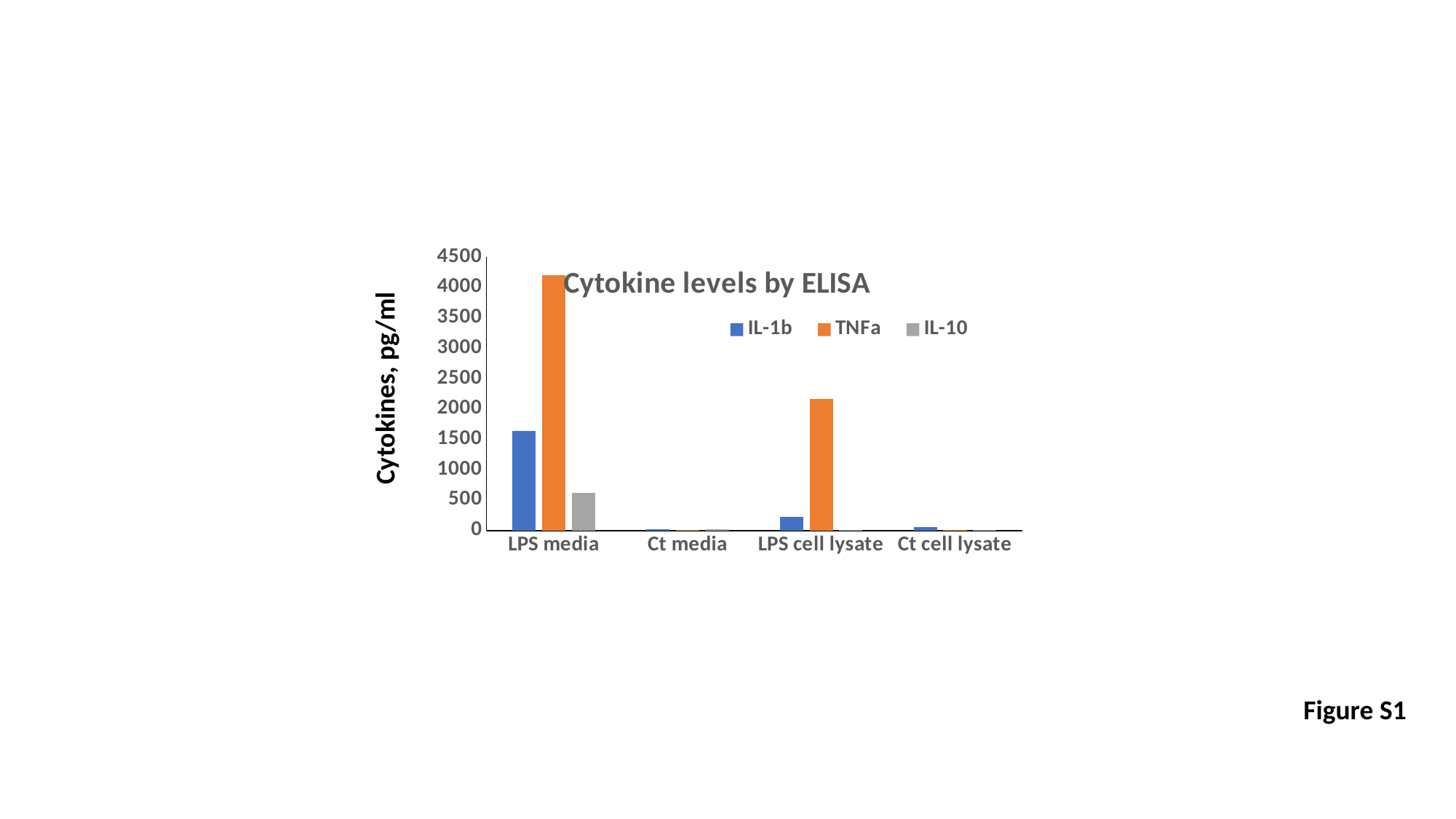

### Chart: Cytokine levels by ELISA
| Category | IL-1b | TNFa | IL-10 |
|---|---|---|---|
| LPS media | 1635.39 | 4202.42 | 625.18 |
| Ct media | 21.51 | 6.46 | 20.37 |
| LPS cell lysate | 224.67 | 2168.15 | 3.57 |
| Ct cell lysate | 54.11 | 7.93 | 5.65 |Cytokines, pg/ml
Figure S1

## Slide 2
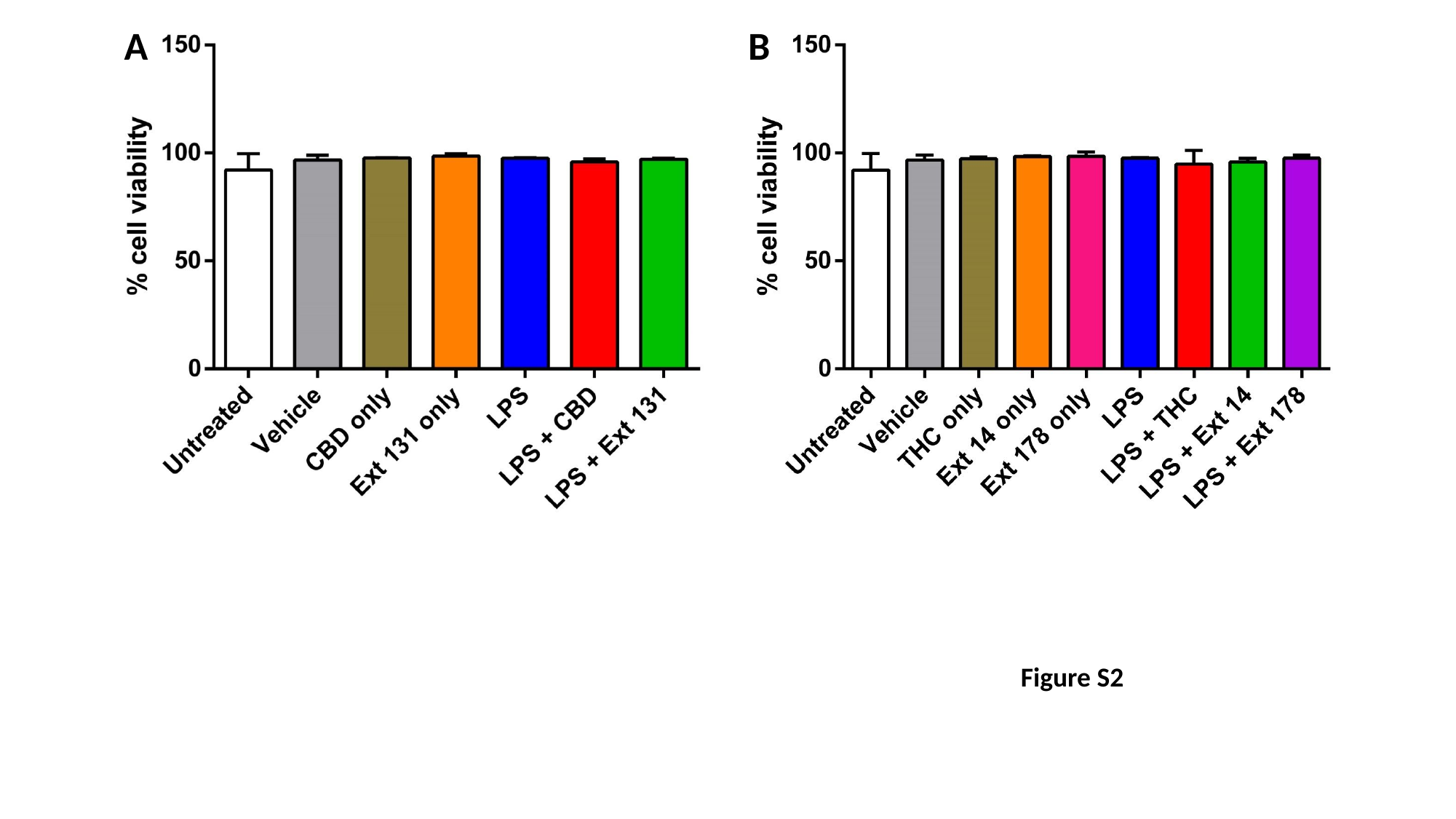

B
A
Figure S2
